# Insights into Canine Idiopathic Rhinitis from a Prospective Study: A Multimodal Diagnostic Perspective

**DOI:** 10.3390/ani16101438

**Published:** 2026-05-08

**Authors:** Sarah Rösch, Gerhard Ulrich Oechtering

**Affiliations:** 1 Ear, Nose and Throat Unit, Small Animal Department, College of Veterinary Medicine, Leipzig University, 04102 Leipzig, Germany; 2 Internal Medicine Service, Small Animal Department, Faculty of Veterinary Medicine, Ghent University, 9820 Merelbeke, Belgium

**Keywords:** allergen-specific IgE, lymphoplasmacytic rhinitis, nasal neoplasia, owner compliance, sinonasal aspergillosis, sinusitis, turbinate destruction

## Abstract

Introduction: Idiopathic rhinitis (IR) is one of the most common causes of chronic nasal discharge in dogs. It is a diagnosis of exclusion requiring a comprehensive diagnostic work-up to rule out other differentials. To investigate a possible allergic etiology, allergen-specific IgE antibody superclasses were evaluated. Methods: Dogs with chronic nasal discharge underwent blood testing, whole-body CT, and detailed evaluation of the nasal cavity. Dogs with systemic diseases or corticosteroid treatment within two weeks prior to presentation were excluded. Results: A total of 46 dogs were included: 13 IR, 10 malignant tumors, six benign tumors, eight other inflammatory nasal diseases, and nine controls. All IR dogs were non-brachycephalic and of various breeds. No correlation was found between sinusitis, turbinate destruction, culture-based bacteriology, or IgE positivity. A similarly high number of positive IgE results was observed across all groups. Regarding treatment and follow-up, compared to dogs with other nasal diseases, compliance among IR dog owners was comparably poor. Conclusions: Allergen-specific IgE may be elevated in dogs with IR; however, they are neither diagnostic nor predictive. As owners often perceive the disease as a harmless cold, awareness must be raised about the nasal functions in dogs and possible consequences of untreated nasal discharge.

## Simple Summary

Idiopathic rhinitis (IR) is a common cause of chronic nasal discharge in dogs, yet its etiopathogenesis remains unclear. Diagnosis relies not only on histopathology of nasal mucosal biopsies but primarily on the exclusion of other nasal, paranasal, and dental diseases through a comprehensive work-up including computed tomography (CT), endoscopy, mycological testing, and histopathology. In this prospective study, dogs with chronic IR were evaluated for sinusitis and turbinate destruction after the exclusion of other conditions by whole-body CT and blood testing. Associations between these findings and culture-based bacteriological results were assessed. Because allergies and fungal spores are frequently proposed as contributing factors, serum allergen screening (superclasses) and Aspergillus antibody results in IR dogs were compared with those of dogs affected by other nasal diseases and healthy controls. No correlation was found between sinusitis, turbinate destruction, and bacteriological culture outcomes in IR dogs. The high rate of positive IgE results in IR dogs was comparable to that observed in other nasal disease groups and controls, without association with the assessed features. Dogs testing positive for IgE classes did not consistently respond to steroid therapy, indicating limited diagnostic and predictive value. Owners of IR dogs showed the lowest compliance and tended to underestimate disease severity.

## 1. Introduction

Canine idiopathic rhinitis (IR) is a diagnosis of exclusion, ideally made after ruling out systemic disease [1], followed by investigations under anesthesia, including head computed tomography (CT), thorough rhinoscopy, negative mycological culture of a nasal mucosal swab, and histopathological confirmation of rhinitis from nasal mucosal biopsies. As demonstrated in an earlier part of this prospective study, routine blood tests show, at most, minor abnormalities and do not significantly differ between dogs with idiopathic rhinitis and those with other nasal diseases. This also applies to ratios, such as the neutrophil-to-lymphocyte ratio and the albumin-to-globulin ratio [2], as well as to standard inflammatory markers such as C-reactive protein (CRP) [3] or commonly used serum tumor markers [4]. An exception is serum survivin [4], which is markedly elevated in dogs with nasal tumors and idiopathic rhinitis, suggesting its potential as a biomarker for therapeutic monitoring; further studies are warranted to confirm its clinical utility. Depending on the study, it ranks among the top three causes of chronic nasal discharge in dogs [5].

IR is often described as a devastating disease, as complete cure is rarely achieved, and treatment often requires individual tailoring [6]. Owners may underestimate its severity compared to the human common cold, leading to limited compliance, despite the fact that dogs are known to be obligate nose breathers [7]. Beside supportive therapy with mucolytic drugs or saline nebulization, reported treatment options include the immune-modulating antibiotic doxycycline [8], corticosteroids [1,9], the immunosuppressive cyclosporine [9], desensitization therapy [9], and, more recently, a protocol of meloxicam followed by prednisolone [10]. Nevertheless, many dogs remain non-responsive [1,9]. Because the etiology of IR is unknown, targeted therapies are currently unavailable.

Culture-based bacteriological examination (cBE) is often positive in affected dogs, more frequently than in other nasal diseases [3]. Recent microbiome studies revealed overrepresentation of pathobionts [11,12] and downregulation of ciliary function genes linked to *Streptococcus* species [11]. It remains unclear whether nasal turbinate destruction in IR correlates with specific bacteria, similar to atrophic rhinitis in swine caused by *Bordetella bronchiseptica* and/or *Pasteurella multocida* [13]. Bacteria are increasingly considered secondary [3], as conventional or even targeted antibiotic therapy rarely results in lasting remission of clinical signs [8]. Doxycycline is effective in some dogs, raising questions about its mechanism of action: either targeting difficult-to-culture bacteria or acting through immunomodulatory effects [14] against possible immune-mediated mechanisms in the etiopathogenesis of IR. However, PCR testing of nasal cavity biopsies from dogs with IR has been negative for *Chlamydia*/*Chlamydophila* spp., *Bartonella* spp., and *Mycoplasma* spp. [15].

Other influencing factors may include disease severity (e.g., involvement of the paranasal sinuses: sinusitis of the frontal, sphenoid sinus or recessus maxillaris; turbinate destruction; and/or secondary otitis media due to Eustachian tube dysfunction/blockage or secondary infection), chronicity of rhinitis, and/or breed predisposition. Commonly affected mesocephalic or dolichocephalic dog breeds (hereafter according to [7] referred to as “normocephalic”, i.e., “non-brachycephalic”) include Jack Russel Terriers, Greyhounds, German Shepherds, and Dachshunds [8,10,16]. This presumed genetic predisposition may relate to the recently reported downregulation of ciliary function genes [11], although affected dogs are typically older [8,10,16].

Although allergic respiratory disease is rare in dogs and the so-called “atopic march” seen in humans [17] is uncommon, some authors still postulate an allergic component involving food or environmental allergens. This aligns with steroid responsiveness and promising results from desensitization therapy in a very small-sized study [9]. Hypersensitivity to airborne *Aspergillus* antigen, known in human medicine as a possible trigger for chronic rhinosinusitis [18], has also been discussed. High levels of fungal DNA in nasal biopsies of dogs with lymphoplasmacytic rhinitis support this hypothesis [15]; however, as *Aspergillus* is a common airborne saprophyte [19], its presence may reflect secondary colonization rather than a primary pathogenic role [15].

This study aimed to describe findings in dogs with IR, focusing on bacterial detection via routine culture as performed in routine clinical settings, and its correlation with nasal turbinate destruction and paranasal sinus involvement detected on CT. We evaluated the presence of serum IgE antibodies against superclasses of indoor and outdoor allergens, comparing dogs with IR to those with other nasal cavity pathologies. Although this screening test does not replace full allergen-specific IgE serology (ASIS) or intradermal testing (IDT) [20], it is commonly used in clinical practice as a screening test for dogs showing relevant clinical signs, before further tests are initiated. By providing detailed information on dogs with nasal disease, we aim to improve owner communication and guide diagnostics. Finally, we investigated whether *Aspergillus* infections or hypersensitivity could play a role in IR etiology, hypothesizing that dogs with IR would show a higher number of positive test results.

## 2. Animals and Methods

### 2.1. Dogs and Study Design

This prospective study with partial blinding was performed as part of an examination of various blood parameters in dogs with nasal discharge due to nasal cavity pathology (animal experiment requiring approval, TV 02/18, Saxony, Germany), after obtaining written owner consent [2,3,4]. Blood was taken prior to anesthesia for diagnostics for nasal cavity disease in fasted dogs. The anesthesia and CT scan were performed as described elsewhere [2]. Beside the CT of the head, a whole-body CT was performed and dogs detected to suffer from additional diseases by CT or blood examination were excluded from the study. Details on excluded dogs have been reported previously [3]. Furthermore, dogs were excluded if they had received corticosteroids (or other immunosuppressives) within 14 days prior to presentation.

As described previously [2,3,4], the diagnosis of nasal cavity disease was made based on the CT scan of the head with contrast medium [2], a detailed anterior and posterior rhinoscopy of the upper respiratory tract [21], and mycological examination of a nasal cavity swab, as well as histopathological examination of biopsies taken of nasal mucosa or nasal tumors under endoscopic view. To ensure comparability, bilateral biopsies were obtained from the ventral turbinates under endoscopic guidance, with the biopsy instrument advanced parallel to the endoscope.

This histopathological evaluation was based on the dominant inflammatory cell type (Antech Lab Germany GmbH, Tierpathologie Munich, Munich, Germany, with Dr. W. von Bomhard, Dipl. ECVP). The following categories were defined: lymphoplasmacytic, necrotizing (characterized by necrotic cellular debris and tissue destruction), neutrophilic, plasmacytic, mycotic, and chronic rhinitis [8]. The latter was defined as inflammatory lesions in combination with remodeling processes (fibrosis, bone remodeling, hyperplasia of the glands, or follicle formation) [8]. Additionally, swabs for cBE were obtained for evaluation.

### 2.2. Grouping of Patients

Based on the examination results, the diagnoses of (1) malignant nasal cavity neoplasia, (2) benign proliferation (patients were repeatedly checked and biopsied to exclude malignant transformation over time), and (3) other inflammatory rhinitis due to a foreign body, periodontopathy or fungal disease (primary or secondary aspergillosis, e.g., secondary to a nasal foreign body) were made. The diagnosis of (4) IR was a diagnosis of exclusion (proposed Tier 3 Confidence Level; Table 1). However, a cBE could be positive in the sense of a secondary bacterial colonization or infection. (5) A control group consisted of nine healthy beagles kept in a habitat at the Faculty of Veterinary Medicine of Leipzig University, in accordance with current and approved guidelines. Their health was confirmed by clinical examination, including the procedures performed under anesthesia as described above. A physical control examination of the control dogs after 4 weeks and 3 months revealed no abnormalities.

### 2.3. Dogs with Idiopathic Rhinitis (IR)

The diagnosis of IR was established by exclusion (proposed Tier 3 Confidence Level; Table 1), including evaluation of blood tests and whole-body CT. Dogs with IR were assessed for the result of the cBE, histopathological examination of nasal mucosa, the presence of sinusitis, and the degree of nasal turbinate destruction on CT (classified as mild-to-moderate or severe). Correlations among these three findings were investigated.

**Figure 1:**
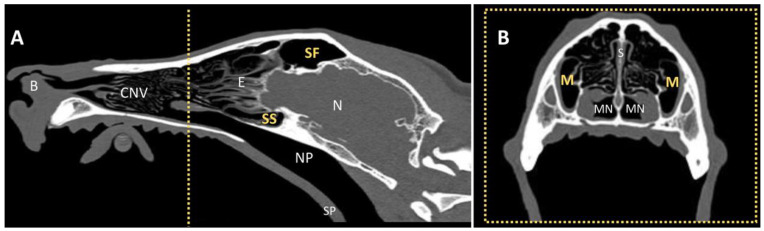
**Paranasal sinuses in the healthy dog: *sinus frontalis* (SF), *sinus sphenoidalis* (SS) and *recessus maxillaris* (M).** (**A**) Longitudinal CT image of the head of a normocephalic (i.e., non-brachycephalic) dog and (**B**) transverse image at the height of the yellow, dotted line in (**A**). B = *Bulbus nasi*, CNV = *concha nasalis ventralis*, E = *conchae ethmoidalis*, NP = nasopharynx, SP = soft palate, N = neurocranium, MN = *meatus nasopharyngeus*.

In contrast to veterinary terminology in dogs, where the terms “idiopathic” or more specifically “lymphoplasmacytic rhinitis” are commonly used, human and feline medicine typically employ the term “rhinosinusitis”, as the paranasal sinuses are often involved in addition to the nasal cavity. Therefore, this study evaluated the frequency of paranasal sinus involvement (*sinus frontalis*, *recessus maxillaris*, and *sinus sphenoidalis*; Figure 1) in dogs with IR using CT. Sinus involvement was defined as filling with soft tissue– or fluid–isodense material on CT. Importantly, each **affected** sinus was subsequently examined endoscopically (after creating endoscopic access if necessary) to confirm fluid accumulation and to rule out fungal granuloma or neoplasia.

### 2.4. Pretreatment in Dogs with IR

**Figure 2:**
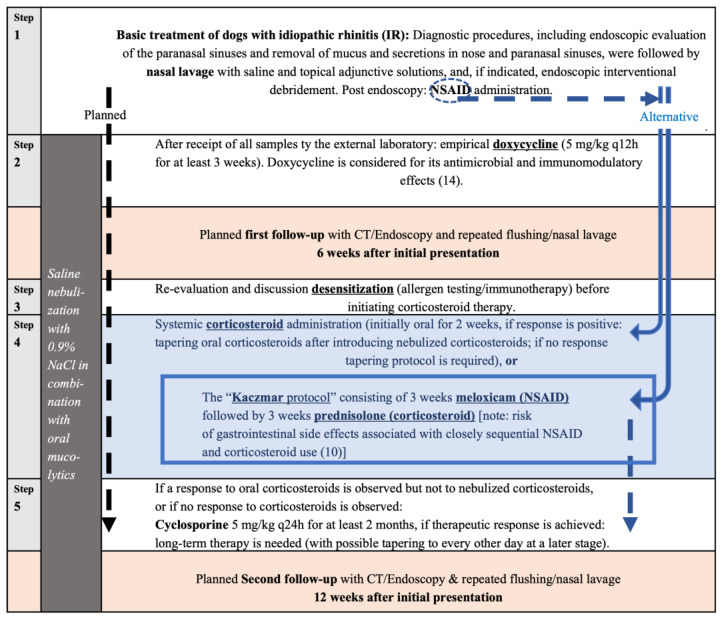
**Stepwise treatment strategy for dogs with idiopathic rhinitis (IR).** Following nasal lavage with saline and topical adjunctive solutions, mechanical debridement via endoscopic intervention under general anesthesia, and NSAID administration after rhinoscopy, a standardized treatment pathway was proposed for this patient cohort (black dashed arrow on the left, labelled “planned”). Treatment recommendations were given based on previous clinical findings and the relevant literature in dogs with IR [8], although the cited study used a higher dosage of doxycycline (up to 10 mg/kg q12h) in some dogs. However, alternative pathways are indicated (blue arrows on the right, either dashed or solid), including the omission of doxycycline when owner consent for the standardized treatment protocol was not given. Except for the ‘Kaczmar protocol’, corticosteroid therapy was initiated only after a short washout period following the short-term NSAID treatment administered after rhinoscopy. In general, at-home therapy with saline nebulization and oral mucolytics was recommended to promote ongoing clearance of mucus and debris. NSAID = non-steroidal anti-inflammatory drug.

As the aim was to complete in all dogs with IR different treatment steps (Figure 2), previously given medications, including antibiotics and corticosteroids, were recorded and evaluated (listed in the Supplementary Table S1).

### 2.5. Allergen-Specific IgE Testing & Aspergillus Antibody Testing

Serological allergen-specific IgE testing, as well as the Aspergillus antibody testing, were performed by an external laboratory (Laboklin, Bad Kissingen, Germany), using an ELISA combined with Fcε-receptor technology in a blinded manner with respect to the patients’ diagnoses (Allercept^®^ serum IgE test, Heska AG, Fribourg, Switzerland). IgE reaction classes ranged from 0 to 5. In line with the majority of previous studies, only reaction classes greater than 3 (i.e., classes 4 and 5) were considered positive in this study, although some studies have classified class 3 as positive [24].

Tested allergen groups included mites (house dust and storage mites), pollen (grass, herb and tree pollen), fungal spores, and flea saliva. Mites and fleas were categorized as indoor allergens, whereas pollen and fungi were classified as outdoor allergens superclasses.

Serological testing for *Aspergillus* antibodies was performed with the Hemkit(R) *Aspergillus* indirect haemagglutination assay (Ravo Diagnostika GmbH, Freiburg, Germany; Laboklin, Bad Kissingen, Germany). Based on the laboratory’s validated reference range, Titers > 1:40 were considered positive and titers < 1:40 negative.

Note: Blood samples were collected at various times throughout the year depending on the patient’s presentation for diagnostics of nasal disease. All examinations were conducted in Leipzig, Saxony, Germany. Therefore, seasonal and regional variation in allergen exposure may have influenced IgE results.

### 2.6. Endoscopy and Endoscopic Intervention

In all dogs of this study, including control dogs, endoscopy was performed as described elsewhere [21] and included anterior and posterior rhinoscopy, as well as endoscopic evaluation of the oral cavity and tonsils, upper esophagus, larynx (including evaluation of functionality), and pharynx. Nasal swabs were collected for further investigations.

In dogs with nasal disease, nasal secretions and mucus were removed under endoscopic visualization using rigid suction tubes in a bimanual approach. Based on CT findings, paranasal sinuses with wider openings, such as the sphenoid sinus and the maxillary recess (*recessus maxillaris*), were evaluated endoscopically, and secretions were suctioned when present. Additionally, all dogs diagnosed with frontal sinus involvement underwent endoscopic sinoscopy [21]. The frontal sinus was opened endoscopically [21], confirming the presence of mucus only and excluding masses or granulomas. The sinus cavity was emptied and irrigated with sterile 0.9% sodium chloride solution (NaCl 0.9%, Braun, Melsungen, Germany).

In diseased dogs, the nasal cavity and affected sinuses were thoroughly flushed with 0.9% sodium chloride, followed by a diluted povidone–iodine solution (Betaisodona^®^, Mundipharma, Limburg, Germany; 1 mL Betaisodona solution per 100 mL 0.9% NaCl) [21], and finally a 0.5% N-acetylcysteine (NAC) solution [25], which has antioxidant, mucolytic, and antibiofilm properties and supports mucociliary clearance [26,27]. The 0.5% NAC solution was prepared from commercially available acetylcysteine ampoules (ACC^®^ Injekt, 100 mg/mL; HEXAL AG, Holzkirchen, Germany) diluted with sterile 0.9% sodium chloride to the target concentration. In the following, both solutions are collectively referred to as topical adjunctive solutions, with antimicrobial properties (povidone–iodine) and antibiofilm/mucolytic properties (NAC). Finally, clotrimazole cream (Canesten^®^, 1% clotrimazole, Bayer, Leverkusen, Germany) was administered endonasally (1–3 mL administered endoscopically, depending on dog size and nasal passage dimensions) to address a possible local irritation or hypersensitivity to topical *Aspergillus* spp. [15]. After endoscopic intervention and biopsy sampling, a non-steroidal anti-inflammatory drug was administered for 3–5 days, depending on the invasiveness of the procedure and according to the discretion of the attending veterinarian.

### 2.7. Treatment and Therapeutic Outcome in Dogs with IR

In all dogs with idiopathic rhinitis, a stepwise treatment protocol was implemented (Figure 2). Previous treatments were documented and discussed with the owner, including whether repetition of prior therapies was considered appropriate. As none of the previously administered treatments had been performed following endoscopic clearance and were often either of insufficient duration or dosage, adherence to the planned treatment algorithm was generally recommended, with possible modifications (e.g., omission of repeat doxycycline if not desired by the owner). Final treatment decisions, including progression through the predefined steps, were made by the owners.

Owners also determined whether follow-up examinations under anesthesia were performed. Repeat CT and rhinoscopy were strongly recommended for all enrolled dogs to evaluate potential disease progression, particularly in cases with poor therapeutic response (e.g., development of additional sinus involvement, bony changes, or otitis media potentially resulting from secondary Eustachian tube dysfunction). These examinations were additionally recommended to allow for an objective assessment of the treatment response.

An initial follow-up under anesthesia was scheduled after completion of Steps 1 and 2, including local flushing and endoscopic intervention, followed by oral medication (e.g., NSAIDs for several days) and a three-week course of doxycycline. A second follow-up examination was scheduled after immunosuppressive therapy (Steps 3 and/or 4 and/or 5). Desensitization therapy was discussed with the owners.

If owners declined follow-up examinations under anesthesia, clinical follow-up was performed via telephone interviews. During these calls, standardized questions based on validated questionnaires [28] were used by ear-nose-throat specialists. When follow-up was based solely on owner opinion, a simple binary scoring system (clinical signs: yes/no) was applied to provide a degree of objectivity, despite the absence of physical re-examination and the known subjectivity of owner-reported clinical signs [28].

### 2.8. Statistics

Statistical analyses were performed using GraphPad Prism (v10.4 GraphPad Software, La Jolla, CA, USA). Data were tested for normality using the D’Agostino and Pearson normality test and the Shapiro–Wilk normality test. Normally distributed data were reported with mean ± SD, while non-parametric data were reported with median and interquartile range. Comparison of parametric data was performed after testing for equality of variance by Brown–Forsythe test and Bartlett test, by one-way ANOVA or between non-parametric data by Kruskal–Wallis test. For the pairwise post-hoc comparison of two parametric data, the unpaired *t*-test was used; for non-parametric data, the Mann–Whitney test was used. To assess contingency, the Chi-square test was applied when more than two categories were present. For comparisons involving two variables, a two-sided Fisher’s exact test was used. A *p*-value < 0.05 was considered significant.

## 3. Results

### 3.1. Dogs of the Present Study & Grouping

The study included 10 dogs with malignant nasal tumors (five carcinomas, five sarcomas), six dogs with benign tumors, eight dogs with benign inflammations, including three dogs with aspergillosis (primary n = 1, secondary n = 2), nine control dogs, and 13 dogs with IR.

In the group of 10 dogs with malignant neoplasia, there were two mixed breeds and one dog of each breed: Golden Retriever, Border Collie, Bolonka Zwetna, Hungarian Vizsla, Harzer Fuchs, Standard Schnauzer, Australian Shepherd, and Weimaraner. 6/10 dogs were male; 4/10 were female. Of these, two male dogs and one female were not neutered. The mean age was 10 ± 2.3 years, and the mean weight was 24.4 ± 10.5 kg. Histopathologically, five carcinomas (two carcinomas, and one each: Adenocarcinoma, carcinoma in situ, low differentiated carcinoma), and five sarcomas (two chondrosarcomas, and one each sarcoma, osteosarcoma, spindle cell sarcoma) were diagnosed. According to the modified T-categories of Adams et al. [29], tumors were assigned according to their CT appearance to T-category 1 (adenocarcinoma, chondrosarcoma), to T-category 2 (adenocarcinoma), to T-category 3 (three chondrosarcomas), and to T-category 4 (carcinoma, transitional cell carcinoma, low differentiated carcinoma, chondrosarcoma).

The group of six dogs with benign tumors included one dog of each breed: Golden Retriever, Labrador Retriever, German Shepherd, Border Collie, French Bulldog, and Silken Greyhound. There were three males, and three females represented. Two males and one female were not neutered. The mean age was 6.2 ± 2.6 years, and the mean weight was 26.6 ± 13 kg. Histopathological examination revealed two cases of polypous rhinitis, two hamartomas, and two cases of benign hyperplasia.

Dogs with other inflammatory diseases (group “others”, n = 8), including three dogs with aspergillosis, one with rhinitis following aspergillosis, two with oronasal defects, and two with foreign objects, comprised four mixed breeds, one Miniature Pinscher, one Pinscher, one Labrador, and one Eurasian. There were seven males and one female. Three of the males and the female were not neutered. The mean age was 7.9 ± 4.1 years, and the mean weight was 22.3 ± 12.4 kg. Histopathology showed chronic rhinitis (n = 3), neutrophilic rhinitis (n = 2), necrotizing rhinitis, plasmacytic rhinitis, and mycotic rhinitis.

The group of control dogs comprised nine dogs of the Beagle breed (four males, five females), which were kept in an outdoor area at Leipzig University. All dogs were neutered. The mean age was 5.8 ± 1.9 years. The mean weight was 11.2 ± 1.2 kg.

There were no significant differences in the mean weight of dogs in different groups. Comparing the age, dogs with malignant nasal tumors were significantly older than the controls (*p* = 0.02).

### 3.2. Dogs with Idiopathic Rhinitis

The group of dogs with IR (n = 13) included three mixed breeds, two Jack Russell Terriers, one Akita Inu, one short-haired Dachshund, one Breton, one Cane Corso, one Appenzeller, one West Highland White Terrier, one Border Collie and one Magyar Vizsla. There were 8/13 male and 3/13 female dogs. Of these, three male dogs and two female dogs were not neutered. The mean age was 6.6 ± 3.3 years, and the mean body weight was 22.4 ± 13.3 kg.

All dogs were normocephalic (i.e., non-brachycephalic). The median duration of clinical signs was 8 months [IQR: 2–18]. Most owners were unable to specify which nasal cavity was affected, although two of 13 reported definitively unilateral nasal discharge. In contrast, diagnostic imaging and endoscopy demonstrated bilateral rhinitis in 12 of 13 dogs, with only one dog showing unilateral disease on CT and/or endoscopy. In two dogs, rhinitis was detected endoscopically but not on CT. Histopathological examination, however, confirmed bilateral rhinitis in all 13 dogs. No bony defects of the nasal bones or lysis of the cribriform plate were detected. Mild septal deviation was observed in three dogs.

The nasal discharge was generally of variable quality, with serous quality in 10/13, mucous in 7/13, and purulent in 13/13. Two of 13 dogs showed epistaxis. Owners reported that 7/13 dogs were sleep-impaired; however, none of the dogs were generally unwell according to the owners.

The cBE was positive in all but five dogs (8/13; 62%; Supplementary Table S1). In contrast, in malignant tumors, benign tumors and dogs of the group “others” was positive in 44%, 14%, and 30%, respectively. Histopathological evaluation of bilateral mucosal biopsies obtained from the ventral turbinates revealed comparable findings on both sides in all 13 dogs, consistent with bilateral rhinitis. Lymphoplasmacytic rhinitis was present in four dogs, chronic rhinitis in five dogs, and neutrophilic in four dogs (Supplementary Table S1). As reported before [3], no correlation between the result of cBE and the type of inflammation was detected.

While 5/13 dogs (38%) showed no involvement of any paranasal sinus, eight out of 13 dogs (62%) were diagnosed with sinusitis (different paranasal sinuses: Figure 1; Supplementary Table S1). Among them, 7/13 dogs had frontal sinus involvement: in 4/7, one frontal sinus and at least one other sinus were affected; in 3/7 dogs, only the frontal sinus was affected; and in 1/7 dogs, only one of the other sinuses (excluding the frontal sinus) was involved. There was no statistically significant association between frontal sinus involvement and the presence of fluid in other sinuses (*p* = 0.27). However, the estimated relative risk for patients with frontal sinus involvement to have additional sinus affection was 2.13 (95% CI: 0.77–6.16). In the 7/13 dogs with frontal sinus affection, 6/7 dogs showed no bony alterations, but presented various fluid patterns: unilateral small amount of fluid in the right frontal sinus; complete filling of the left frontal sinus, up to 70% filling of the left frontal sinus with fluid level and air bubbles, complete filling of the right frontal sinus with dorsal air bubbles, and a fluid level in the left sinus frontalis. In 1/7 dogs, bony alteration (new bone formation) was observed in addition to the bilateral complete filling of the frontal sinuses. The remaining paranasal sinuses (*sinus sphenoidalis* and/or *recessus maxillaris*) were affected in 5/13 dogs (38%), with two dogs showing involvement of both the *sinus sphenoidalis* and the *recessus maxillaris*. Of the five dogs, three dogs had unilateral fluid-filled sphenoid sinuses (two on the right side, one on the left); four dogs showed a fluid level in the maxillary recess (two bilateral, two left-sided).

Among the eight dogs with sinusitis, cBE of a nasal swab was positive in four cases (50%). Similarly, a positive cBE was found in four out of five dogs without sinusitis (80%). There was no statistically significant association between positive bacterial culture and the presence of sinusitis (*p* = 0.57).

Nasal turbinate destruction was identified by CT and endoscopy in 7/13 dogs, with three cases classified as mild and four as severe. Despite two dogs with turbinate destruction showing *ß-hemolytic Streptococcus*, no correlation was found between the presence of nasal turbinate destruction and the bacteria detected at the time of diagnosis (e.g., *Pasteurella multocida* or *Staphylococcus aureus*; *p* = 0.59).

Turbinate destruction was observed in six of eight dogs with sinusitis (62.5%) and in one of five dogs without sinusitis (20%). Although sinusitis was more frequent among dogs with turbinate destruction (5/6, 83.3%), the association was not statistically significant (*p* = 0.27). The estimated relative risk was 2.0 (95% CI: 0.88–5.57).

### 3.3. Pretreatment

During the period of clinical signs (median 8 months [IQR: 2–18]), all 13/13 dogs had been pretreated with one or more antibiotics, as reported before [3]. Five of 13 dogs had been pretreated with corticosteroids at varying and in some cases partly unknown dosages without improvement in clinical signs.

### 3.4. Endoscopic Intervention

**Figure 3:**
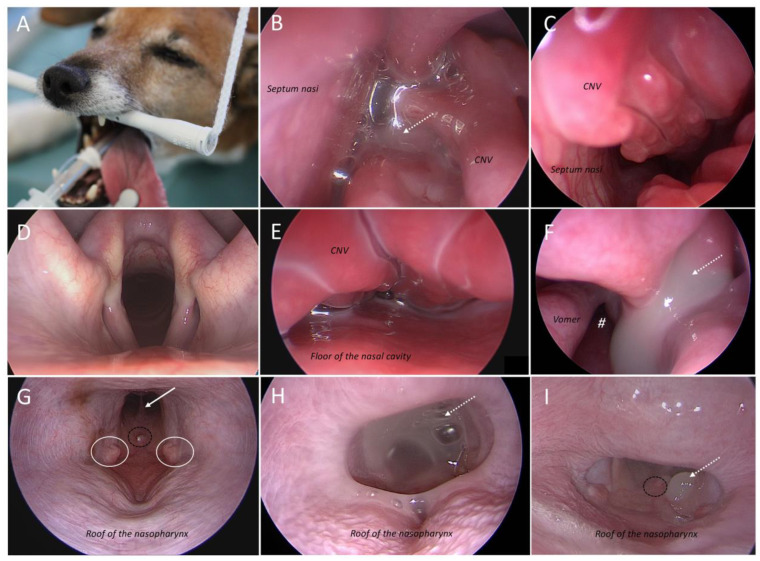
**Endoscopic images of dogs with idiopathic rhinitis included in the study.** (**A**) Optimal positioning of a patient for upper airway endoscopy (without prior lavage) using a modified maxillary sling (Patient No. 6). (**B**) Endoscopic view of the nasal cavity (Patient No. 42) during examination without lavage, showing the characteristic serous to whitish discharge (dashed white arrow). This illustrates the authors recommendation to suction nasal secretions in every patient with rhinitis to allow complete endoscopic evaluation of the nasal cavity. (**C**) Endoscopic image of Patient No. 6, who showed mild turbinate destruction of the left *concha nasalis ventralis* (CNV) on CT, also demonstrates endoscopic findings suggestive of ‘turbinate destruction,’ characterized by an increased and abnormal air space between the turbinate branches. This appearance suggests that so-called turbinate destruction does not necessarily reflect true structural loss, but may also result from altered apposition or partial collapse of the turbinate structures. (**D**) Example of a physiologically normal larynx (Patient No. 45). In addition to evaluation of the larynx (including movement), the tympanic membranes/middle ears, trachea up to the main bronchi, and the proximal esophagus were endoscopically examined in all patients. (**E**) Another example of mild turbinate destruction. Again, the remaining turbinate branches of the CNV show the absence of normal spacing between structures. (**F**) Nasal cavity exit (#) with whitish, viscous discharge (dashed white arrow) draining from the ethmoidal turbinates (Patient No. 6). (**G**–**I**) Images obtained using a 120° optic, resulting in a reversed orientation of the nasopharynx. Visible structures include the roof of the nasopharynx, the auditory tube openings (white circles in G), and the nasal septum (white arrow in G). Black circle in G: rostral margin of the pharyngeal tonsil with a small proliferative lesion (*BIPS = BenIgn nasopharyngeal Proliferative Structure*). (**G**) Patient No. 24. (**H**) Patient No. 10: nasopharynx obstructed by mucoid discharge (dashed white arrow). (**I**) As seen in Patient No. 42, the discharge (dashed white arrow) may appear highly viscous, explaining the clinical presentation of reverse sneezing.

All dogs underwent endoscopy of the upper airways (Figure 3) and endoscopic intervention for nasal cleaning, including removal of mucus in all 13/13 dogs. All dogs were flushed with diluted povidone–iodine in addition to 0.9% NaCl, and 9/13 dogs were additionally flushed with diluted acetylcysteine as a mucolytic and biofilm-disrupting agent. In 9/13 dogs, in the end of the procedure, 1% clotrimazole cream was additionally applied into the nasal cavity (not into frontal sinuses; Canesten^®^, 1% clotrimazole, Bayer, Leverkusen, Germany, 2–4 mL per side depending on the size of the nasal cavity; Supplementary Table S1). Following diagnostic evaluation, an NSAID was administered for 3–5 days or longer if clinically indicated (Supplementary Table S1).

In none of the 13 dogs did the clinical signs of nasal disease completely resolve after endoscopic intervention, including the cases in which the sinuses were opened and/or flushed. A stepwise therapeutic plan, as outlined in Figure 2, was implemented in all dogs, and follow-up re-evaluations using CT and endoscopy were recommended, since clinical signs do not always accurately reflect disease status in nasal cavity disorders.

### 3.5. Allergen-Specific IgE Tests

**Figure 4:**
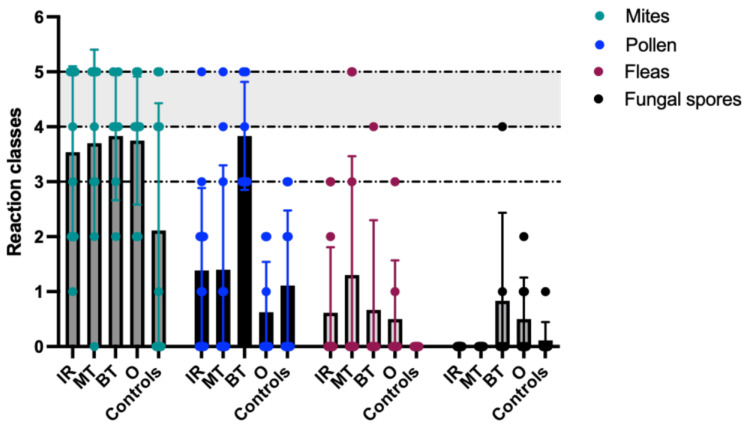
**IgE reaction classes in dogs with the different nasal cavity pathologies to mites, pollen, fungal spores, and fleas.** Idiopathic rhinitis (IR) n = 13, malignant tumor (MT) n = 10, benign tumor (BT) n = 6, other inflammatory conditions (O) n = 8, controls n = 9. Mite-specific IgE was detected at reaction classes above 3 in the majority of dogs across all disease groups but less frequently in controls. Pollen-specific IgE levels were particularly elevated in dogs with benign tumors compared to the other groups.

Of the 46 dogs included in the study, including control dogs, the serum allergen screening test was positive in 28/46 dogs (61%) when only reaction classes 4 and 5 were considered positive (Figure 4). An additional five dogs showed reaction class 3, which has been classified as positive in previous studies [24]. Using this threshold, 33/46 of the dogs (72%) would be considered positive.

Of the 28 dogs with reaction classes 4 and 5, 24 dogs (86%) had nasal cavity pathology, while four dogs (14%) belonged to the control group. All 28 positive dogs were positive for at least one allergen group. In 21/28 dogs, only one allergen group was positive, predominantly mites (20/21), with one dog reacting solely to pollen. In 5/28 dogs, two allergen groups were positive (mites + pollen in four dogs, mites + fleas in one dog). In 2/28 dogs, three allergen groups were positive (mites + pollen + fleas in one dog and mites + fungi + flea in the other). All 28 dogs showed elevated indoor allergen levels. Seven dogs additionally reacted to outdoor allergens, and only one dog tested positive for fungal spores.

As mite-specific IgE (reaction class 4 and 5) were the most common, seven out of 13 dogs (54%) with IR tested “positive”. Comparable or higher “positivity rates” were observed in other groups: 6/10 dogs (60%) with malignant tumors; 4/6 (66%) with benign tumors; 6/8 (75%) of the group others; and 4/9 (44%) in the control group (Figure 4). Fungal spore-specific IgE was rare, detected in only one of six dogs (17%) with a benign tumor, while all other dogs tested negative for this allergen group. Flea-specific IgE was identified in only three dogs: two with malignant tumors (20%) and one with a benign tumor (17%). Pollen-specific IgE was detected in 1/13 dogs (8%) with IR, 2/10 dogs (20%) with malignant tumors, and 3/6 dogs (50%) with benign tumors.

### 3.6. Aspergillus Antibody Test

Serological testing for *Aspergillus* antibodies was available in 12 of 13 dogs with IR. Overall, the test was positive in five dogs across all groups: 2/12 dogs (17%) with IR, despite negative fungal culture and histopathology; 2/10 dogs (20%) with malignant nasal tumors; and 1/9 dogs (11%) in the control group. The control dog had no clinical signs, unremarkable CT and endoscopic findings, and negative fungal culture and histopathology. Notably, all dogs with confirmed primary or secondary aspergillosis tested negative.

### 3.7. Oral Medication at Home and Follow-Up

**Figure 5:**
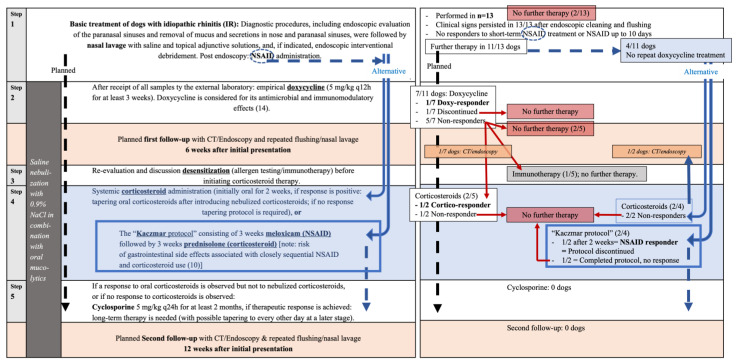
**Initially proposed stepwise treatment protocol (Figure 2), illustrating how the treatment pathways were actually followed based on the owners’ decisions (in the mirrored right-hand panel, summarizing the outcomes of each step).** While only seven dogs followed the planned pathway, four of the 11 dogs that remained in treatment deviated to an alternative pathway, as repeat doxycycline therapy was not desired. On the right: Responders are indicated in bold black: one dog responded to doxycycline, one to a 14-day NSAID course, and one to corticosteroids. Only two dogs underwent follow-up examination—one after doxycycline therapy and one after corticosteroid administration. Based on the owner’s decision, none of the patients reached step 5 or received cyclosporine, and none of the dogs underwent a second follow-up examination with CT/endoscopy.

As described previously, all included dogs underwent a stepwise therapeutic protocol (Figure 2 and Figure 5). Although this medication was discussed with the owner, and follow-up evaluations were recommended and potential complications of persistent nasal discharge explained, not all owners followed the treatment steps and only two of 13 dogs with IR (15%; Supplementary Table S1) were presented for follow-up examination under anesthesia. In contrast, among the 10 dogs diagnosed with malignant tumors, six of eight dogs (75%) were presented for follow-up examinations under anesthesia (two dogs were euthanized during diagnostics at the owners’ request). Of those six dogs, five had a single follow-up examination, and one had four follow-up examinations. Only two of the 10 dogs did not undergo any follow-up examinations.

All owners of IR dogs were instructed to administer daily nebulization therapy using 0.9% saline solution for at least 15 min per day. Additionally, oral acetylcysteine (ACC) was prescribed as a mucolytic agent (Supplementary Table S1; Figure 5).

**Treatment Step 1**—Following endoscopic intervention, including slime removal, nasal lavage, and short-term post-procedural NSAID administration (Step 1; Figure 2), clinical signs persisted in all 13 dogs.

Further medical therapy was initiated in 11 of 13 dogs, following endoscopic debridement and after all diagnostic results were available. In two of 13 dogs, owners declined further treatment immediately after diagnostics, as despite participating in this prospective trial, their primary concern had been to rule out neoplastic, mycotic, or dental disease and foreign object.

**Extended Treatment Step 1—NSAID**: In two of the 11 dogs (18%), owners declined additional antibiotic therapy, as the dogs had previously been treated with multiple distinct antibiotics. Both dogs were therefore treated with prolonged meloxicam following endoscopic intervention in regard to the treatment protocol of Kaczmar et al. [10], consisting of three weeks of NSAID followed by three weeks of prednisolone (see Step 4). This protocol will hereafter be referred to as the ‘Kaczmar protocol’. However, one dog showed complete resolution of clinical signs after two weeks of the NSAID meloxicam alone (NSAID responder, Step 1, Supplementary Table S1), and the owner discontinued all medication. The second dog completed the anti-inflammatory protocol described by Kaczmar et al. [10] (see Step 4), but showed no clinical improvement after completion, and the owners declined any further therapy.

**Treatment Step 2—Doxycycline**: Seven of 13 dogs had received doxycycline prior to referral (four dogs for three weeks, two dogs for one week, and one dog twice for an unknown duration), without clinical improvement. Following endoscopic intervention (Step 1), owners agreed upon oral doxycycline in seven of 11 dogs, in which further medical therapy was initiated. Regarding prior treatment, doxycycline treatment was repeated in two cases (one dog having been previously treated for three weeks and one dog having been previously treated for one week). Among the 7/11 dogs receiving doxycycline after completion of diagnostics, 5/7 (71%) were classified as non-responders and showed no clinical improvement. One dog (14%) developed gastrointestinal adverse effects, resulting in discontinuation of therapy after five days. Only 1/7 (14%) achieved complete resolution of clinical signs following doxycycline therapy administered after endoscopic intervention and NSAID treatment.

**Treatment Step 3—Desensitization therapy:** Only one dog received an unspecified immunotherapy despite negative supergroup testing, with minimal clinical improvement; all other owners declined specific desensitization therapy.

**Treatment Step 4—Corticosteroids and ‘protocol of Kaczmar’ [10]:** Four of 13 IR dogs had received corticosteroids prior to diagnostics without clinical improvement (three orally and one via nebulization). After completion of diagnostics, four dogs were treated with corticosteroids, including the one dog following the complete ‘Kaczmar protocol’ (as described above). Three of these dogs, including the one following the ‘Kaczmar protocol’, showed no clinical response, whereas one achieved complete resolution of clinical signs. Therefore, in total, seven dogs received corticosteroids (either prior to or after diagnostics) and only 1/7 responded (14%).

Among the dogs receiving corticosteroids, five dogs had positive allergen-specific IgE serology; only one demonstrated clinical improvement. Two IgE-negative dogs were treated with corticosteroids, and neither responded. Fisher’s exact test revealed no significant association between positive IgE status and clinical response to corticosteroid therapy (*p* > 0.99).

**Treatment Step 5—Cyclosporine:** None of the dogs in this study were treated with cyclosporine.

**In conclusion**, as shown in Figure 5 and the Supplementary Table S1, the treatments were highly variable. Three dogs responded to therapy: one to doxycycline, one to NSAID, and one to corticosteroids. Of the remaining 10 dogs, five owners declined further therapy after a lack of response to the initial treatments, while the other five declined further therapy and were lost to follow-up.

## 4. Discussion

This study provides a multifaceted evaluation of dogs diagnosed with IR, combining advanced imaging, endoscopic assessment, and immunological and microbiological diagnostics. Despite the small cohort size (n = 13) resulting from the exclusion of dogs with other/systemic diseases (confirmed via blood tests and whole-body CT) and/or prior corticosteroid treatment, the findings offer valuable insights into the diagnostic complexity and therapeutic challenges associated with IR in dogs.

Dogs included in the IR group were exclusively normocephalic (i.e., non-brachycephalic), which is beneficial for study comparability. Brachycephaly is known to cause anatomical alterations in the nasal cavity, such as an increased number of mucosal contact points [22] and airflow dynamics, which may predispose dogs to rhinitis and impact the efficacy of medical therapies. Additionally, brachycephalic dogs often lack frontal sinuses, making them a distinct clinical subgroup that should be investigated separately.

Any paranasal sinus involvement (*sinus frontalis*, *sinus sphenoidalis*, or *recessus maxillaris*) was observed in 8/13 dogs (62%) on CT, most frequently affecting the frontal sinuses (7/8 dogs). Sinusitis was confirmed in all affected cases by endoscopic interventional opening or access to the involved sinus. Because this study evaluated all paranasal sinuses, the overall rate of sinus involvement (62%) is slightly higher than in studies that assessed only the frontal sinuses, and which reported frontal sinus involvement in approximately 42% of cases [30]. This supports that the term “rhinosinusitis” may be a more appropriate term than “rhinitis” in dogs, as it is already used in both feline and human medicine.

The presence of turbinate destruction in more than half of the dogs with IR (7/13, 54%) is noteworthy. Although the association between turbinate destruction and sinusitis did not reach statistical significance (*p* = 0.27; relative risk = 2.0), a trend toward a higher prevalence of sinusitis in dogs with turbinate destruction was observed. This finding is particularly relevant in dogs in whom only rhinoscopic examinations can be performed due to owner limitations, as careful attention to the outflow tracts of the paranasal sinuses is required to allow for endoscopic trephination when secretions indicate sinusitis. Endoscopic trephination and flushing of the sinuses did not result in complete disease resolution in the dogs of this study. However, the procedure of sinus trephination was essential to exclude fungal or neoplastic disease. It may also have helped slow disease progression and improve quality of life, given that nonpolypoid sinusitis in humans is frequently associated with moderate severe headache, facial pain, and pressure within the paranasal sinuses [31].

While turbinate destruction is classically associated with fungal rhinitis (e.g., *Aspergillus* spp.), our findings support the presence of an idiopathic rhinosinusitis subgroup with turbinate destruction, which has previously been reported in up to 70% of cases [30]. However, grading of turbinate destruction and sinus involvement remains subjective, and more standardized scoring systems are needed. In this study, a simple binary grading system with “yes/no” and “mild/severe” was used, to reduce influence of subjective evaluation.

Culture-based bacteriological examination (cBE) results were evaluated despite the known limitations of cBE, such as false negatives due to prior antibiotic use or fastidious organisms, and false positives from contaminants. Modern alternatives like next-generation sequencing (NGS) could improve sensitivity and specificity in future studies [11,32]. However, cBE is displaying conditions in daily clinical practice. This study showed no significant association between cBE result and sinusitis or turbinate destruction. The duration of infection, the timing of sampling, and the cultivability of the pathogens may have played a role but cannot be reliably determined due to the chronic nature of the disease.

In dogs with IR, the influence of an environmental allergy is frequently discussed. Beside corticosteroids and cyclosporine, desensitization is a described treatment option [9] and may be considered when dogs exhibit elevated IgE levels. However, IgE screening alone is not diagnostic and must be interpreted within the broader clinical context [33]. To evaluate whether dogs with IR achieve positive test results more often than other groups, which could be helpful in making a diagnosis or in informing prognosis for treatment with steroids or a desensitization therapy, IgE antibody levels (allergen superclasses) were investigated in dogs with IR in comparison to other groups of dogs with nasal cavity pathologies. In this study, 61%, or nearly two-thirds, of dogs tested positive when only reaction classes 4 and 5 were considered positive. Specifically, 54% of IR dogs exhibited positive IgE results—similar rates to dogs with malignant (60%) and benign (66%) nasal tumors. The highest reaction classes were found against environmental allergens such as mites. However, it is striking that all dogs with benign nasal tumors were positive for outdoor allergens. No correlation was found between positive IgE screening results and response to corticosteroids, highlighting the complexity of interpreting such tests in IR cases. Findings from other veterinary studies (e.g., atopic dermatitis) report comparable seroprevalence (71%) in the serum screening test [34]. A study by Baumann and colleagues showed that differences in the results between different laboratories lead to reduced reproducibility of the tests [33]. However, it should be emphasized once again that this initial screening test does not replace full allergen-specific IgE serology (ASIS) or intradermal testing (IDT) [20]. The Allercept^®^ serum IgE assay, validated for dogs and applied in the present study, has been shown to demonstrate variable diagnostic performance when compared with intradermal testing, with results differing depending on the specific allergen investigated [35]. Despite the exclusion of dogs with other diseases detected on whole-body CT, blood examination, or clinical examination, which could have affected the test results, conditions that are more difficult to detect with these modalities, such as endoparasites or the administration of drugs other than immunosuppressants, could not be fully ruled out. Therefore, a potential residual effect cannot be excluded. However, as IgE analysis did not reveal significant differences between groups and was not associated with a relevant clinical treatment response to corticosteroids (in cases where it was performed), we consider it unlikely that any residual confounding substantially influenced the overall interpretation of the results.

*Aspergillus* serology was included in the diagnostic work-up, as this study also aimed to investigate whether testing for Aspergillus antibodies is warranted in dogs with IR, given that sensitivity to this pathogen may play a role in mycologically negative cases, as observed in some humans with chronic rhinosinusitis [18]. However, no significantly higher number of positive cases was detected compared to other groups. Additionally, despite confirmed *Aspergillus fumigatus* infections in three dogs (one with primary aspergillosis and two with secondary aspergillosis due to nasal foreign bodies), none showed elevated antibody titers. This reinforces the limited diagnostic sensitivity of serological approaches including the indirect haemagglutination assay used in this study and agar gel immunodiffusion, while ELISA-based assays generally show higher sensitivity with variable diagnostic performance despite high specificity [36,37]. Thus, negative serology cannot exclude fungal disease, and diagnosis of aspergillosis should continue to rely on a combination of imaging, mycological examination of nasal mucosa swabs, and histopathology.

Therapeutic outcomes were difficult to assess scientifically, as most dogs had already undergone multiple treatment attempts prior to presentation and only 11 of the 13 dogs received medical therapy following endoscopy.

During endoscopy, the nasal cavity was cleaned and irrigated in all dogs. As only short-term improvement was observed in most cases, the value of endoscopic mucus removal and extensive lavage may be questioned. These procedures prolong anesthesia time and require careful airway protection (e.g., placement of swabs alongside the endotracheal tube in the pharyngeal area), as well as advanced endoscopic skills, including bimanual coordination. However, it should be emphasized that nasal cleaning and irrigation have a major benefit in detecting small tumors, foreign objects, or subtle abnormalities within the nasal cavity. Therefore, despite the limited therapeutic effect, we consider these procedures a standard component of a thorough endoscopic examination.

Furthermore, as stated above, drainage of secretions from the paranasal sinuses via an endoscopically created access route did not provide therapeutic benefits. However, this procedure remains strongly recommended for diagnostic purposes in case of sinusitis, as fungal granulomas might otherwise remain undetected [38].

The topical administration of 1% clotrimazole cream in dogs is generally well tolerated and was performed for addressing either hypersensitivity to fungi leading to IR as described in humans (primary disease) or in response to increased fungal load in the nasal mucus due to idiopathic rhinitis (secondary). However, no difference in clinical outcome was observed between treated and untreated dogs, nor with respect to whether the nasal cavity was additionally flushed with diluted iodine solution and acetylcysteine. Importantly, no worsening of clinical signs of nasal disease was noted. In contrast, owners reported a temporary improvement following the procedure; however, this finding requires further evaluation using dedicated follow-up assessments with validated questionnaires. Therefore, while we recommend acetylcysteine as a nasal cleansing step beside diluted iodine solution, we do not recommend routine application of clotrimazole cream, and further studies are warranted to clarify its efficacy.

In many cases, after step 1 of therapy, only one additional level of treatment (Figure 2; e.g., doxycycline) could be initiated, as further escalation was limited by owner consent. Follow-up imaging or rhinoscopic re-evaluation was performed only in two cases, as the remaining owners did not present their dogs for further follow-up, which limits the ability to objectively assess treatment response. Owner perception of IR often differs from the clinical reality [28]. In contrast to malignant diseases (see high number of follow-ups in dogs with malignant nasal tumors), the condition is frequently perceived as ‘benign’—comparable to a human cold—leading to lower compliance and reluctance toward long-term treatment with antibiotics, corticosteroids, or immunosuppressants. However, IR in dogs can impair nasal function and predispose them to complications such as postnasal drip, aspiration pneumonia, otitis media, and potentially sinus pain, as reported in human medicine [39,40]. It should be emphasized that the nose has important functions, and loss of nasal function can significantly impair quality of life in dogs.

This study again demonstrates that idiopathic rhinitis (IR) is a disease that is difficult to treat. In a previous study [8], 1/17 dogs with IR (17%) showed no clinical nasal signs after endoscopic intervention and short-term NSAID therapy. Importantly, follow-up was based solely on owner reports without imaging re-evaluation. In this study, none of the dogs responded to short-term NSAID treatment; however, one dog showed a positive response after a two-week course of NSAIDs. Furthermore, only one dog responded to corticosteroid therapy. Due to the heterogeneous treatment allocation and small subgroup sizes in both treatment groups, percentage-based response rates were not considered meaningful for treatment with NSAIDs or corticosteroids in this study.

In the previously cited study [8], approximately 24% of dogs with IR showed a treatment response to doxycycline administered for 3 weeks; again, the absence of clinical signs was based solely on owner reports, without imaging re-evaluation. In the present study, only one dog (1/7) responded to doxycycline therapy (14%). In contrast, we report this response as a percentage, as all seven dogs received this treatment, and a defined denominator is available. This allows a limited comparison with previously published data and provides an estimate of the potential response rate to doxycycline following diagnostic work-up and treatment, although this study represents a tertiary referral population. This implies that response rates in less selected populations or earlier stages of referral may be higher.

In contrast to reports in the literature, and in line with the observations of the authors and the outcome in one dog from this study, the ‘Kaczmar protocol’ [10] did not result in clinical improvement. However, as this study shows, individual dogs may respond to prolonged NSAID or corticosteroid therapy, which is why this approach was included in our stepwise protocol. We note, however, that steroid responders typically require long-term therapy rather than the three-week course described.

The therapeutic effect of corticosteroids in dogs with IR remains inconsistent. In this study, only one of three treated dogs showed a clear clinical response. When considering prior treatments, five dogs with positive allergen-specific IgE serology received corticosteroids, with only one responding clinically. Additionally, none of the two IgE-negative dogs showed improvement. These findings suggest that a positive IgE status alone may not predict therapeutic success with corticosteroids in dogs with IR.

Several limitations must be acknowledged. The sample size was small, and subgroup analysis was limited by group size imbalance reducing statistical power. The study population may have been biased due to the referral nature of the institution, and no standardized treatment protocol was used across all patients due to a low owner compliance. No repeated CT/rhinoscopy weakened the evaluation of therapeutic response, despite a binary scoring system being used for clinical signs to reduce subjective interpretation of disease status by the owners. Seasonal variation and environmental exposure could not be controlled. It should be noted that the two-week corticosteroid withdrawal period prior to examination is considered sufficient in some reports, while others recommend a longer interval (e.g., 28 days for injectable glucocorticoids) [41]. Therefore, the influence of withdrawal duration on our results cannot be fully excluded. Finally, as noted above, the outcome of the serological allergen-specific IgE testing as a screening tool do not replace full allergen-specific IgE serology (ASIS) or intradermal testing (IDT) [20].

## 5. Conclusions

This study demonstrates that sinusitis, turbinate destruction, positive cBE, and positive IgE classes can occur in dogs with IR, but that there is no correlation between these findings or with specific bacterial species. IgE positivity can be observed in all dogs with various nasal diseases, and a positive test result is not helpful in diagnosing IR or predicting whether a dog with IR will respond to corticosteroids, in the sense of an underlying immunological etiology. Terms such as “rhinosinusitis” or “rhinosinusitis with turbinate destruction” may help better describe the findings in individual patients, as well as the confidence levels for the diagnostic work-up proposed in this study in Table 1. A larger number of dogs with IR after exclusion of concurrent diseases based on the extensive diagnostic work-up performed in this study, as well as better owner compliance with the planned treatment protocol, could have provided more comprehensive information on this patient population. However, a clearer understanding of the etiopathogenesis of IR remains necessary. Treatment can therefore be challenging. However, owners must be informed that this is not a harmless “cold”, as untreated cases can significantly impair quality of life. Various treatment attempts are justified to achieve relief of clinical signs and to prevent secondary complications.

## Figures and Tables

**Table 1:** **Updated [22] and modified [23] confidence levels for the diagnosis of idiopathic rhinitis (IR).** Adapted from the tiered confidence levels originally established for the diagnosis of idiopathic epilepsy [23]. These levels are designed to be referenced during the diagnostic process, allowing clinicians to assign a diagnostic category and estimate the certainty of the diagnosis “IR”.

Tier 1 Confidence Level	Signalment and breed predisposition. Chronic nasal discharge—assessment of duration and characteristics of the discharge (unilateral vs. bilateral, quality). Minimum database: Blood work to rule out systemic diseases (e.g., leishmaniasis, ehrlichiosis). No blind sampling of nasal discharge is recommended, because: ○ Bacterial isolates are not significantly different between disease entities. ○ Detection of *Aspergillus* spp. in mycological examination may represent either primary or secondary sino-nasal aspergillosis (SNA) [21], requiring further diagnostics.	Note: Only a **presumptive diagnosis** is possible at this stage, as various nasal diseases may present with similar clinical signs. Additionally, dental disease may affect only the tooth roots, which may not be visible during clinical and dental examination.
Tier 2 Confidence Level	Includes all Tier 1 components, plus: **Anterograd and retrograde rhinoscopy**—allows detection of nasal masses, foreign bodies, parasites, turbinate destruction, fungal granulomas in the nasal area accessible during standard rhinoscopy, and evaluation of the opening of the frontal sinus (detection of pathological fluid discharge as an indicator of sinusitis). **Mycological examination:** Due to its high rate of false-negative results, mycological testing should ideally be performed in conjunction with histopathology, since fungal elements may also be visualized histologically. **Histopathological examination of nasal mucosa**—enables evaluation of the type of inflammation and helps to detect fungal disease. ***Culture-based bacteriological examination (cBE): of limited clinical utility and questionable role [3,8].***	Note: Paranasal sinus disease (neoplasia; aspergillosis) and apical dental pathology may still be missed at this level of diagnostics. The cBE did not assist in diagnosing nasal disease or guiding therapy [3,8] and may therefore lead to a clinical dilemma when results are positive. Its role in the management of IR remains questionable.
Tier 3 Confidence Level	Includes all Tier 1 and Tier 2 components, plus: **Computed Tomography** (CT)—provides detailed evaluation of sinus involvement, detection of dental pathology, otitis media, bony alterations (e.g., cribriform plate lysis), or other lesions not easily accessible via endoscopy.	Note: As malignant nasal neoplasia is the most common cause of chronic nasal discharge and prognosis worsens with increasing tumor size, early diagnostics with a Tier 3 confidence level are recommended.

## Data Availability

The datasets used and/or analysed during this study are available from the corresponding author upon reasonable request.

## Supplementary Materials

The following supporting information can be downloaded at: https://www.mdpi.com/article/10.3390/ani16101438/s1, **Supplementary Table S1. Diagnostic findings in dogs with idiopathic rhinitis (IR). Only positive test results for pollen, fungal, mites, and flea antigens (including the reaction group) and antibodies against *Aspergillus* spp. (titer > 1:40) are reported.** Orange fields indicate turbinate destruction; green indicates frontal sinus involvement; blue indicates involvement of other sinuses; yellow indicates a positive result in culture-based examination (cBE). Dark grey indicates prior corticosteroid therapy (dosage unknown; n.a. = not available); light pink indicates a positive antigen test; light grey indicates treatment responders (doxycycline, n = 1; NSAID, n = 1); and dark pink indicates response to corticosteroid therapy after diagnostics (n = 1).

## Abbreviations

ASIS	Allergen-specific IgE serology
cBE	Culture-based bacteriological examination
CT	Computed tomography
IDT	Intradermal testing
IR	Idiopathic rhinitis
M	*Recessus maxillaris*
NSAID	Non-steroidal anti-inflammatory drug
SF	*Sinus frontalis*, frontal sinus
SS	*Sinus sphenoidalis*, sphenoid sinus
