# Supplementary material for: Insights into Canine Idiopathic Rhinitis from a Prospective Study: A Multimodal Diagnostic Perspective
Source: Animals (Basel). 2026 May 8;16(10):1438. doi: 10.3390/ani16101438 (PMC13203159; doi:10.3390/ani16101438)
Supplement: Supplementary file 1 [file animals-16-01438-s001.zip › animals-4227803-supplementary.pdf]

Supplementary Table S1. Diagnostic findings in dogs with idiopathic rhinitis (IR). Only positive test results for pollen, fungal, mites, and flea antigens (including the reaction group) and antibodies against *Aspergillus* spp. (titer > 1:40) are reported. Orange fields indicate turbinate destruction; green indicates frontal sinus involvement; blue indicates involvement of other sinuses; yellow indicates a positive result in culture-based examination (cBE). Dark grey indicates prior corticosteroid therapy (dosage unknown; n.a. = not available); light pink indicates a positive antigen test; light grey indicates treatment responders (doxycycline, n=1; NSAID, n=1); and dark pink indicates response to corticosteroid therapy after diagnostics (n=1).

	**CT-based evaluation**						**History**		**Blood test**	
IR-Dog No. in prospective study	Uni- or bilateral?	Nasal turbinate destruction?	Affection of frontal sinus?	Affection of other paranasal sinuses	Result of culture-based bacteriological examination (cBE)	Histo-pathology	Antibiotic pretreatment (dosage n.a.) = **without improvement**	Cortisone pretreatment (dosage n.a.) = **without improvement**	Antigen group	During diagnostics the following topical treatments were applied: B (povidone iodine), ACC (topical acetylcysteine), C (clotrimazole cream); Subsequent treatment was initiated after completion of diagnostics and endoscopic cleaning
1	Bilateral	No	No	No	*Pasteurella multocida*	Bilateral lympho-plasmacytic	Different antibiotics, last one: amoxicillin clavulanic acid for four weeks	Oral corticosteroids for one week	**Mites 5/5,** flea 2/5, pollen 1/5	• B, C<br>• Oral robenacoxib[1], oral acetylcysteine[2], once-daily nebulization with 0.9% saline solution for 15 minutes.<br>• Oral doxycycline[3] (9 mg/kg q24h for 21 days), combined with nicotinamide (niacin, vitamin B3) 250 mg q12h; no improvement of clinical nasal signs.<br>• Further treatment declined.<br>• Dog died due to causes unrelated to disease.<br><br>**NON-RESPONDER NSAID and Doxycycline *[pre-report:***

[1] Onsior®, Elanco Animal Health, Bad Homburg, Germany; 2 mg/kg q24h subcutaneously, followed by 1 mg/kg q24h orally for 5 days.

[2] ACC®, 200 mg powder (Hexal, Holzkirchen, Germany), 3–5 mg/kg q8–12h orally.

[3] Doxy-M-ratiopharm®, 100 mg tablets (Ratiopharm, Ulm, Germany).

										corticosteroids] – Step 1 and 2 and [4]
6	Bilateral	Low grade	Right frontal sinus filled	Fluid level maxillary recess bilateral	*Pasteurella canis*	Chronic rhinitis	Doxycycline for three weeks (according to antibiogram)	Corticosteroid injection	Mites 2/5	• B, ACC<br>• Oral robenacoxib[1] and amoxicillin 12.5 mg/kg q12h for 5 days, oral acetylcysteine[2], and once-daily nebulization with 0.9% saline solution for 15 minutes.<br>• Doxycycline[3] for 21 days: partial improvement.<br>• Immunotherapy (not further specified by the owner/veterinarian) also resulted only in partial improvement.<br>• Further treatment declined.<br><br>**NON-RESPONDER NSAID and Doxycycline *[pre-treatment corticosteroids] – Step 1 and 2 and [4]***
9	Bilateral	No	No	No	*Streptococcus canis*	Chronic rhinitis	Amoxicillin clavulanic acid for three weeks	Oral Corticosteroids for two weeks	**Mites 5/5**, pollen 2/5	• B, ACC, C<br>• Meloxicam[4] for 5 days, oral acetylcysteine[2], once-daily nebulization with 0.9% saline solution for 15 minutes.<br>• Doxycycline[3]: partial improvement only.<br>• Further treatment declined.<br><br>**NON-RESPONDER NSAID and Doxycycline [pre-report *corticosteroids] – Step 1 and 2 and [4]***
10	Bilateral (left> right)	Caudal nasal cavity, ethmoidal	Left frontal	Fluid filled left sphenoid	*ß-hemolytic streptococcus*	Neutrophilic	Enrofloxacin for eight		**Mites 5/5**, pollen 2/5,	• B, ACC, C

[4] Metacam®, 1.5 mg/mL suspension (Boehringer Ingelheim, Ingelheim am Rhein, Germany), initially 0.2 mg/kg q24h subcutaneously, followed by 0.1 mg/kg q24h orally.

		turbinates, low grade	sinus filled	sinus, fluid level left maxillary recess			weeks (according to antibiogra m)		*asper-gillus* 1:40	• Robenacoxib[1] for 5 days, oral acetylcysteine[2], once-daily nebulization with 0.9% saline solution for 15 minutes.<br>• Doxycycline[3] discontinued after 5 days due to gastrointestinal adverse effects.<br>• Further treatment declined. **No corticosteroids** (discouraged by owner and veterinarian) and further treatment declined.<br>• Homeopathic therapy initiated by the owner.<br><br>**NON-RESPONDER NSAID and Doxycycline – *Step 1 and 2***
18	Bilateral	No	70% filled with fluid level	No	Negative	Chronic rhinitis	Unknown –over three months		Mites 1/5, pollen 2/5	• B, ACC, C<br>• Once-daily nebulization with 0.9% saline solution for 15 min<br>• Meloxicam[4] for 21 days, followed by 21 days of **prednisolone** according to the protocol of Kaczmar (10): no clinical improvement.<br>• Further treatment declined.<br><br>**NON-RESPONDER NSAID, and prednisolone – *Step 2, 4***
23	**Uni-lateral**	Yes, high grade	No	No	*Staphylococcus aureus*	Lympho-plasmacyti c	Amoxicilli n clavulanic acid for one week		Mites 2/5, pollen 3/5	• B, C<br>• Meloxicam[4] for 10 days, oral acetylcysteine[2] and once-daily nebulization with 0.9% saline solution for 15 minutes.<br>• Doxycycline[3] for 21 days.

										**RESPONDER DOXYCYCLINE – *Step 2***
24	No alteration in CT – only bilateral in endoscopy	No	No	No	Negative	Chronic rhinitis	Doxycycline for one week		**Mites 5/5**, pollen 2/5	• B, C<br>• Further treatment declined.<br><br>***Lost to follow up***
26	No alteration in CT – only bilateral in endoscopy	No	No	No	*S. intermedius*	Lympho-plasmacytic	Amoxicillin clavulanic acid for two weeks		Mites 2/5	• B, ACC, C<br>• Meloxicam[4] for 14 days, oral acetylcysteine[2], and once-daily nebulization with 0.9% saline solution for 15 minutes<br>• Complete resolution of clinical signs.<br><br>**RESPONDER NSAID – *Step 1***
29	Bilateral	Yes, high grade bilateral	Both frontal sinus fluid filled	Fluid filled right sphenoid sinus	*ß-hemolytic streptococcus*	Lympho-plasmacytic	Doxycycline two times, amoxicillin clavulanic acid, unknown duration		Mites 3/5, flea 3/5	• B, ACC, C<br>Meloxicam[4] for 10 days, oral acetylcysteine[2], once-daily nebulization with 0.9% saline solution for 15 minutes.<br>• Further treatment declined.<br><br>***Lost to follow up***
42<br><br>Dog with follow-up CT/endoscopy/histopathology	Bilateral	No	No	Fluid level left maxillary recess	*Pasteurella multocida*	Chronic rhinitis	Doxycycline for three weeks		Mites 2/5, pollen 1/5	• B, ACC<br>• Robenacoxib[1] for 3 days, oral acetylcysteine[2], once-daily nebulization with 0.9% saline solution for 15 minutes.<br>• **Followed by Prednisolone** 1 mg/kg q24 hours – no clinical improvement.<br>• Further treatment declined.<br><br>***Lost to follow up***

										**NON-RESPONDER NSAID, [pre-treatment Doxycycline] and prednisolone – *Step 1, [2], 4***
45<br><br>Dog with follow-up CT/endoscopy/histopathology	Bilateral	Yes, starting in the middle of the nasal cavity	Yes, fluid level left frontal sinus	No	Negative	Bilateral Neutrophilic	Cefalexin for unknown duration, doxycycline for one week		**Mites 5/5**	• B, ACC<br>• Meloxicam[4] for 14 days, oral acetylcysteine[2] and once-daily nebulization with 0.9% saline solution for 15 minutes.<br>• Doxycycline[3] for 21 days: **no clinical improvement.**<br>• **Inhaled corticosteroid therapy: marked clinical improvement.**<br><br>**RESPONDER STEROID NEBULIZATION – step 4**
47	Bilateral	Yes, medium grade, especially caudal ethmoidal turbinates	Yes complete filling right frontal sinus, left frontal sinus small amount with fluid level	Fluid filled right sphenoid sinus, bilateral fluid level in maxillary recess (right> left)	Negative	Bilateral Neutrophilic	Doxycycline for three weeks	Nebulization with 0.9% NaCl-Dexamethasone - solution	**Mites 4/5**	• B, C<br>• **Oral prednisolone** 1,25mg/kg/d for 14 days, oral acetylcysteine[2], once-daily nebulization with 0.9% saline solution for 15 minutes.<br>• Owner did not fully adhere to treatment recommendations; Further treatment declined.<br><br>***Lost to follow-up.***<br><br>**NON-RESPONDER NSAID, Doxycycline *and corticosteroids – Step 1, 2 and 4***
53	Bilateral (left> right)	Yes, ethmoidal turbinates, low grade	Complete filling left frontal sinus	No	Negative	Bilateral Neutrophilic	Amoxicillin clavulanic acid for two weeks	Oral corticosteroids for 4 days	**Mites 5/5, pollen 5/5,** *Aspergillus* 1:320	• Meloxicam[4] for 5 days, oral acetylcysteine[2], once-daily nebulization with 0.9% saline solution for 15 minutes.<br>• Doxycycline[3] for 21 days<br>• **Oral corticosteroids:** No clinical improvement.

										• Further treatment declined.<br><br>***Lost to follow up***<br><br>**NON-RESPONDER NSAID, Doxycycline *and corticosteroids – Step 1, 2 and 4***
